# TECHNOLOGY-BASED ASSESSMENT OF EMOTIONS AND SOCIAL BEHAVIORS IN OLDER ADULTS’ AND COUPLES’ HOMES

**DOI:** 10.1093/geroni/igad104.1833

**Published:** 2023-12-21

**Authors:** Kuan-Hua Chen, Robert Levenson

## Abstract

As people age, emotions and close social interactions are increasingly important because our social circle tends to shrink. Research on social and emotional aging typically relies on questionnaires and laboratory assessments. However, most emotions and social behaviors occur in naturalistic and interpersonal contexts. Advances in modern technologies, including telecommunications, mobile or embedded devices and sensors, provide researchers with exciting opportunities for naturalistic, long-term, objective, and timely assessment of emotions and social behaviors outside of the lab. Increased accessibility of technologies further allows community-based, large-scale assessment. The proposed symposium highlights recent advances in-home assessment of emotions and social behaviors from four different institutions that leverage various active (e.g., ecological momentary assessment, portable lab) and passive (i.e., motion and door sensors, wearables) technologies to assess multiple aspects of emotions and social behaviors (e.g., physiological responses, emotional experience, behaviors and interpersonal behavioral synchrony, apathy, agitation) in healthy and clinical aging. Four presentations covering four different exemplary studies will be given by researchers at different career stages with diverse ethnic/cultural and training backgrounds (e.g., mechanical engineering, psychology, neuroscience). An integrated discussion will be given by a senior researcher and leading expert in the area. Together, the symposium will provide the general audience with an overview of recent advances in in-home assessment technologies. This symposium will also launch communication and collaborations between researchers from different disciplines who use (or plan to adopt) contemporary technologies to conduct research in naturalistic settings to promote a better understanding of human social and emotional aging.

